# 33 Setting the Standard: Using a National Burn Registry to Benchmark Risk Adjusted Mortality

**DOI:** 10.1093/jbcr/irac012.036

**Published:** 2022-03-23

**Authors:** Samuel P Mandell, Sara Higginson, Kimberly Hoarle, Naiwei Hsu, Darryl A Pardon, Bart D Phillips, Matthew H Phillips, Callie M Thompson, Joan M Weber, Palmer Q Bessey

**Affiliations:** UT Southwestern, Parkland Regional Burn Center, Dallas, Texas; UC San Diego Health Regional Burn Center, San Diego, California; American Burn Association, Chicago, Illinois; Torrance Memorial Medical Center, Torrance, California; BData, Inc, Louisville, Kentucky; BData, Inc, Edina, Minnesota; BData, Inc, Apex, North Carolina; University of Utah Health, Salt Lake City, Utah; Shriners Hospitals for Children Boston, Boston, Massachusetts; New York-Presbyterian Hospital / Weill Cornell Medicine, New York, New York

## Abstract

**Introduction:**

Reports of single center experience and studies of larger databases have identified several predictors of burn center mortality, including age, burn size, and inhalation injury. None of these analyses has been broad enough to allow benchmarking across burn centers. The purpose of this study was to derive a reliable risk-adjusted, statistical model of mortality based on real-life experience at many of the burn centers in the United States.

**Methods:**

We used a national burn registry to identify 128,252 initial admissions from July 2015 through June 2020 across 103 unique burn centers. We selected 23 predictor variables, from over 50 recorded in the dataset based on completeness (at least 75% complete required) and clinical significance. Missing data were multiply imputed with a Bayesian Ridge Regression estimator. All data analysis was performed in Python using Numpy and Scikit-Learn libraries. We used Gradient boosted regression (CatBoost), a form of machine learning, to predict mortality and compared this to traditional logistic regression. Model performance was evaluated with AUC and PR curves. Using the CatBoost predictions, observe to expected mortality was calculated for each center. Confidence intervals for O/E analysis in the case of mortality prediction were calculated using a custom implementation of the Clopper-Pearson method. Analyses were run on three cohorts: All patients; Patients with 10-20% TBSA; and >20% TBSA.

**Results:**

The CatBoost model achieved a test AUC of 0.982 with an average precision of 0.801. The logistic regression, by comparison, produced an AUC of 0.974 with an average precision of 0.726. While accuracy, the measure most reported in the literature, is near ceiling for both models, the CatBoost model is markedly more sensitive, leading to a substantial improvement in average precision. Because of the superiority of the CatBoost model with respect to outcome prediction, we only used CatBoost models for calculation of O/E ratio (Fig. 1).

**Conclusions:**

Gradient boosted regression models provided greater model performance than traditional, multivariate, logistic regression. Using data from a national burn registry, we can predict burn mortality across contributing centers allowing for meaningful O/E ratios. Further, this allows for comparison of mortality across centers contributing data to the registry.

